# Repeated inoculation of cattle rumen with bison rumen contents alters the rumen microbiome and improves nitrogen digestibility in cattle

**DOI:** 10.1038/s41598-017-01269-3

**Published:** 2017-04-28

**Authors:** Gabriel O. Ribeiro, Daniela B. Oss, Zhixiong He, Robert J. Gruninger, Chijioke Elekwachi, Robert J. Forster, WenZhu Yang, Karen A. Beauchemin, Tim A. McAllister

**Affiliations:** 1 Lethbridge Research and Development Centre, Agriculture and Agri-Food Canada, Lethbridge, AB, T1J 4B1 Canada; 2 Departamento de Zootecnia, Universidade Federal de Viçosa, Viçosa, MG, 36570-900 Brazil

## Abstract

Future growth in demand for meat and milk, and the socioeconomic and environmental challenges that farmers face, represent a “grand challenge for humanity”. Improving the digestibility of crop residues such as straw could enhance the sustainability of ruminant production systems. Here, we investigated if transfer of rumen contents from bison to cattle could alter the rumen microbiome and enhance total tract digestibility of a barley straw-based diet. Beef heifers were adapted to the diet for 28 days prior to the experiment. After 46 days, ~70 percent of rumen contents were removed from each heifer and replaced with mixed rumen contents collected immediately after slaughter from 32 bison. This procedure was repeated 14 days later. Intake, chewing activity, total tract digestibility, ruminal passage rate, ruminal fermentation, and the bacterial and protozoal communities were examined before the first and after the second transfer. Overall, inoculation with bison rumen contents successfully altered the cattle rumen microbiome and metabolism, and increased protein digestibility and nitrogen retention, but did not alter fiber digestibility.

## Introduction

The rumen microbiome consists of a complex microbial community composed of bacteria, archaea, protozoa, and fungi. The metabolic activity of these microbial symbionts converts complex fibrous substrates into volatile fatty acids (VFA) and microbial protein that are used by the ruminant host for maintenance, growth and lactation^1^. Although the rumen is one of the most effective systems for degrading plant cell walls, less than 50% of cell wall carbohydrates are digested in low quality forages such as straw^2^. Improving the efficiency of structural carbohydrate degradation in the rumen would provide additional energy for animal production at a substantial value to the beef and dairy industries.

Bison may be more efficient at digesting low-quality forages (<7% crude protein) than cattle^3–5^. A hypothesis as to why the rumen microbiome in bison is more efficient at digesting plant cell walls is that it co-evolved to digest the natural high-lignocellose feedstuffs consumed by bison^6^. When fed similar high-forage diets, the bison rumen microbiome has been shown to have a superior fiber-digesting capacity to beef cattle as it has more fibrolytic bacteria (i.e., *Fibrobacter succninogenes*, *Ruminococcus albus* and *Ruminococcus flavefaciens*)^7^, differs in protozoal species, and has greater total protozoal numbers^8^.

The introduction of pure cultures of fibrolytic bacteria into the ruminal microbiome to enhance fibre degradation has been largely unsuccessful, possibly due to the resilience and host-specificity of this complex microbial community^9^. In addition, most pure fibrolytic bacterial cultures have been maintained for many years in the laboratory and grown under controlled laboratory conditions where they may become less competitive and comparably less fit than their wildtype counterparts within the host^10–12^. Competition for substrates with fully adapted indigenous rumen bacteria may account for the failure of these inoculated cultures to integrate into the complex rumen microbial community.

Rumen protozoa may represent up to 50% of microbial biomass and play a key role in ruminal N and carbohydrate metabolism^13^. In contrast to bacteria, protozoa have been shown to have very little host specificity^14–16^. The importance of the rumen protozoa in fibre degradation of high forage diets has been demonstrated by comparing protozoa-free sheep to faunated sheep^17, 18^. The effect of the manipulation of protozoa on rumen metabolism without their complete elimination (defaunation) is not completely understood^17^ and the introduction of specific protozoa populations into the faunated rumen as a means of improving fibre degradation has not been investigated.

Rumen content transfer across ruminant species has not been described and may be a means of overcoming the limits in fitness imposed by laboratory culture methods. Hence, the objective of this proof-of-concept study was to assess if the fibre digestibility of a barley straw-based diet was enhanced as a result of the transfer of rumen contents from bison to cattle. We hypothesised that the capacity of cattle to digest barley straw would be improved as a result of the transfer of rumen contents from bison to cattle. Two rumen transfers were conducted to increase the likelihood that microbes responsible for the degradation of the most recalcitrant fibre in the bison rumen had the opportunity to establish in the rumen of heifers.

## Results

No animal health issues were encountered during the entire 18 week experiment. Bison rumen contents substituted 72.4% ± 1.80% and 71.0% ± 2.23% (DM basis) of the heifer rumen contents during the first and second transfer, respectively. The body weight (mean ± SD, kg) of heifers increased from 447 ± 26.7 kg to 475 ± 24.1 kg over the duration of the experiment (data not shown). However, the average daily gain of heifers did not differ between before vs. after transfers of rumen contents. Ruminal pH, total numbers of protozoa and the proportion of *Isotricha* within the protozoal population were numerically lower in ruminal contents from heifers as compared to bison (Table 1).

**Table 1:** Chemical composition, pH and protozoa population of heifers’ rumen contents before rumen transfers and from the bison inoculum used in the rumen transfers.

Item	Before 1^st^ rumen transfer		Before 2^nd^ rumen transfer	
	Heifers	Bison inoculum^1^	Heifers	Bison inoculum^1^
Rumen DM content, kg	5.5 ± 1.08	—	5.5 ± 0.89	—
pH	6.76 ± 0.093	7.25	—	7.19
Protozoa				
Total, ×10^4^	13.4 ± 4.28	34.4		28.2
*Entodinium*, %^2^	83.3 ± 7.70	85.0	—	79.8
*Ostracodinium*, %^2^	7.2 ± 6.69	1.8	—	3.9
*Metadinium*, %^2^	2.9 ± 3.56	1.2	—	0.0
*Polyplastron*, %^2^	2.3 ± 2.59	0.0	—	0.0
*Isotricha*, %^2^	1.7 ± 2.34	7.4	—	10.6
*Eudiplodinium*, %^2^	0.9 ± 1.17	0.7	—	2.1
*Diplodinium*, %^2^	0.8 ± 1.39	0.7	—	1.4
*Dasytricha*, %^2^	0.5 ± 0.93	2.5	—	1.4
*Ophryoscolex*, %^2^	0.2 ± 0.51	0.2	—	0
*Epidinium*, %^2^	0.1 ± 0.35	0.3	—	0.7
Chemical composition				
DM, %	9.5 ± 0.84	13.2	9.1 ± 0.98	12.0
OM, % of DM	87.0 ± 2.01	87.3	86.6 ± 2.22	85.7
CP, % of DM	12.6 ± 0.98	11.1	13.3 ± 0.86	14.4
NDF, % of DM	68.7 ± 2.74	73.6	67.9 ± 2.47	69.9
ADF, % of DM	44.6 ± 2.17	52.1	40.9 ± 1.36	39.7

DM, dry matter; OM, organic matter; CP, crude protein; NDF, neutral detergent fiber; ADF, acid detergent fiber.

^1^From pooled samples of all the bison rumen contents used.

^2^% of total protozoa count.

### Intake, digesta kinetics, gut fill, and total tract digestibility

Dry matter intake (kg) increased (*P* < 0.05) after rumen transfers on a daily as well as on a percentage of BW and a BW^0.75^ basis (Table 2). Although intake was increased after transfers, the fractional passage rate of solids and fluids did not differ (*P* > 0.10) between period, with the exception that the delay to first marker appearance in the fluid phase was longer (*P* = 0.02) after rumen transfers. Gut fill (total DM in gut) increased (*P* ≤ 0.02) after rumen transfers. The rumen transfers did not affect (*P* ≥ 0.44) apparent total tract digestibility of DM, OM, NDF or ADF, but it did increase N digestibility (*P* < 0.01).

**Table 2:** Feed intake, digesta kinetics and total tract digestibility of heifers fed a barley straw diet before and after rumen content transfers from the bison.

Item	Rumen transfers		SEM	*P*-value
	Before	After		
Feed intake				
DM, kg/d	6.22	7.14	0.166	<0.01
DM, % of BW	1.39	1.50	0.042	0.04
DM, g/kg of BW^0.75^	64.1	70.3	1.88	<0.01
OM, % of BW	1.30	1.40	0.032	0.04
NDF, % of BW	0.93	0.99	0.029	0.03
digested NDF, % of BW	0.48	0.51	0.019	0.37
Digesta kinetics^1^				
Solids				
Ks, %/h	2.22	2.10	0.097	0.26
TD, h	11.6	10.1	0.86	0.11
RMRT, h	47.0	48.3	2.09	0.57
FMRT, h	6.82	7.13	0.261	0.32
TMRT, h	65.4	65.5	2.35	0.98
Fluids				
Ks, %/h	5.16	4.94	0.194	0.14
TD, h	11.2	8.5	0.91	0.02
RMRT, h	19.9	20.6	0.79	0.31
FMRT, h	2.42	3.41	0.617	0.24
TMRT, h	33.5	32.5	0.73	0.21
Total DM in gut, kg	12.5	14.3	0.52	0.01
Total DM in gut, % of BW	2.68	3.01	0.094	0.02
Apparent total tract digestibility, %				
DM	52.6	52.9	0.85	0.79
OM	55.2	55.0	0.87	0.89
NDF	51.8	50.9	1.25	0.55
ADF	48.6	47.3	1.22	0.44
N	63.3	66.5	0.72	<0.01

DM, dry matter; OM, organic matter; NDF, neutral detergent fiber; ADF, acid detergent fiber; N, nitrogen.

KS (%/h) = ruminal passage rate; TD (h) = time delay to first marker appearance.

RMRT (h) = ruminal mean retention time; FMRT (h) = first compartment mean retention time.

TMRT (h) = total tract mean retention time = RMRT + FMRT + TD.

^1^Etimated by the nonlinear model with gamma-4 age dependency in the fast compartment (G4G1).

### Nitrogen utilization and ruminal microbial protein synthesis

After rumen transfers, N intake increased (*P* < 0.01), along with fecal and urinary N losses (*P* < 0.01; Table 3). Even with increased fecal and urinary losses, the increase in N intake and apparent total tract N digestibility (*P* < 0.01) resulted in an increase (*P* < 0.03) in the amount of retained N (g/d) as a result of rumen transfers. The flow of rumen microbial N (g/d) increased after rumen transfers (*P* = 0.03), but the efficiency of microbial N production (g/kg of digested OM) was not affected (*P* ≥ 0.77).

**Table 3:** Nitrogen (N) utilization and ruminal microbial protein synthesis by heifers fed a barley straw diet before and after rumen content transfers from the bison.

Item	Rumen transfers		SEM	*P*-value
	Before	After		
N intake, g/day	151	179	3.8	<0.01
Fecal N, g/day	55	60	1.5	<0.01
Urine N, g/day	90	108	2.7	<0.01
Retained N (RN), g/day	5.3	13.2	2.57	0.03
RN/N intake, %	3.2	7.2	1.53	0.07
RN/digested N, %	4.9	10.6	2.34	0.09
RN, g/kg of BW^0.75^	0.054	0.113	0.0310	0.14
Total purine derivatives, mmol/day	100.5	110.1	3.41	0.02
Allantoin, mmol/day	94.8	103.4	3.07	0.02
Uric acid, mmol/day	5.7	6.7	0.49	0.06
Microbial purines absorbed, mmol/day^1^	74.2	83.4	3.96	0.03
Microbial N flow, g/day^1^	53.9	60.6	2.88	0.03
Microbial N flow, g/kg of BW^0.75^	0.556	0.597	0.0249	0.17
Microbial N, g/kg of DOM^2^	16.9	16.7	0.84	0.77

^1^Estimated as described by Chen and Gomes^64^ using the urine purine derivative excretion. ^2^DOM, digested organic matter.

### Ruminal pH profile and fermentation characteristics

**Figure 1:**
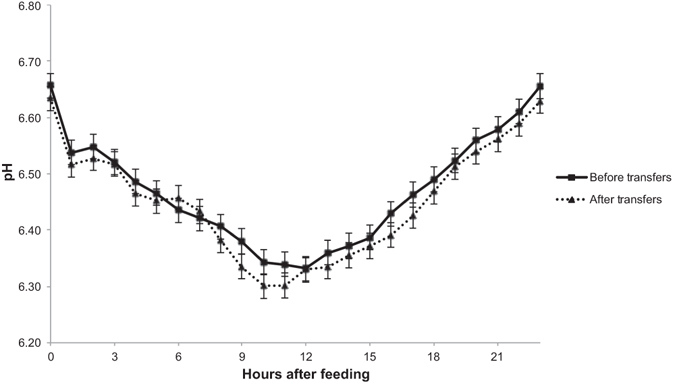
Mean daily ruminal pH of heifers fed a barley straw diet before and after rumen content transfers from the bison (5 days of measurement). No treatment or treatment × time interaction (*P* > 0.05) was observed.

Although small, a reduction (*P* ≤ 0.03) in mean and maximum pH was observed after rumen transfers (Table 4; Fig. 1). After rumen transfers, the pH was also lower at 6 h post feeding. Ruminal NH_3_-N (m*M*) before feeding was not affected (*P* = 0.77) by rumen transfers, but was higher (*P* = 0.05) 6 h after feeding. After rumen transfers, total VFA (m*M*) and the molar proportion of butyrate and valerate increased (*P* < 0.01) prior to and at 6 h after feeding, whereas the proportion of acetate and the C2:C3 ratio decreased (*P* ≤ 0.02) prior to and at 6 h after feeding. After rumen transfers isovalerate and isobutyrate proportions were higher (*P* < 0.01) before feeding, but were not affected (*P* ≥ 0.13) 6 h after feeding.

**Table 4:** Ruminal pH profile of fermentation characteristics of heifers fed a barley straw diet before and after rumen content transfers from the bison.

Item	Rumen transfers		SEM	*P*-value
	Before	After		
Ruminal pH				
Mean	6.47	6.45	0.005	<0.01
Minimum	6.11	6.07	0.040	0.17
Maximum	6.76	6.74	0.016	0.03
SD of mean pH	0.14	0.14	0.007	0.88
Immediately before feeding				
pH	6.70	6.66	0.025	0.27
Ammonia-N, m*M*	5.52	5.59	0.206	0.77
Total VFA, m*M*	88.8	104.7	2.37	<0.01
Acetate, mol/100 mol	74.7	72.5	0.27	<0.01
Propionate, mol/100 mol	18.2	18.5	0.20	0.24
Butyrate, mol/100 mol	5.50	6.68	0.150	<0.01
Isovalerate, mol/100 mol	0.95	1.04	0.022	<0.01
Valerate, mol/100 mol	0.38	0.48	0.018	<0.01
Isobutyrate, mol/100 mol	0.68	0.75	0.013	<0.01
Acetate:Propionate	4.10	3.93	0.058	0.02
6 h after feeding				
pH	6.24	6.07	0.039	<0.01
Ammonia-N, m*M*	7.80	8.80	0.461	0.05
Total VFA, m*M*	106.4	123.0	3.08	<0.01
Acetate (C2), mol/100 mol	71.2	69.6	0.27	<0.01
Propionate (C3), mol/100 mol	20.1	20.4	0.173	0.17
Butyrate, mol/100 mol	6.81	8.02	0.124	<0.01
Isovalerate, mol/100 mol	0.64	0.59	0.028	0.13
Valerate, mol/100 mol	0.65	0.85	0.022	<0.01
Isobutyrate, mol/100 mol	0.57	0.55	0.013	0.24
Acetate:Propionate	3.55	3.42	0.042	<0.01

After rumen transfers total protozoa counts and the proportion of *Ostracodinium* increased (*P* < 0.01), whereas *Entodinium* decreased (*P* < 0.001) before and at 6 h after feeding (Table 5). Post ruminal transfer, there was also a tendency for the proportion of *Metadinium* to be lower (*P* ≤ 0.07) before and 6 h after feeding. Numbers of *Polyplastron*, *Isotricha, Dasytricha* and *Epidinium* were consistently low and often absent so as a result only their prevalence was reported. After rumen transfers, the prevalence of *Epidinium* increased (*P* < 0.01) both before and at 6 h after feeding. There was a tendency for a greater (*P* = 0.06) prevalence of *Dasytricha* before feeding after rumen transfers. The proportion of *Isotricha* was higher in rumen digesta from bison than from heifers, but transfer of bison digesta did not increase *Isotricha* within the rumen contents of heifers.

**Table 5:** Ruminal protozoa population of heifers fed a barley straw diet before and after rumen content transfers from the bison.

**Item**	Rumen transfers		SEM	*P*-value		
	Before	After		Time	Transfer	Time × Transfer
Total Protozoa, ×10^4^						
Before feeding	7.5	10.0	0.55	0.02	<0.01	0.67
6 h after feeding	6.4	8.5				
*Entodinium*, %^1^						
Before feeding	73.1	57.2	2.77	0.35	<0.01	0.23
6 h after feeding	67.8	57.9				
*Ostracodinium*, %^1^						
Before feeding	7.2	21.2	1.88	0.35	<0.01	0.41
6 h after feeding Item	7.0	18.2				
*Eudiplodinium*, %^1^						
Before feeding	5.5	5.6	1.04	0.02	0.69	0.32
6 h after feeding	7.2	7.9				
*Metadinium*, %^1^						
Before feeding	6.7	5.8	1.00	0.24	0.07	0.51
6 h after feeding	8.2	5.6				
*Diplodinium*, %^1^						
Before feeding	4.9	4.6	1.16	0.71	0.27	0.32
6 h after feeding	4.8	4.9				
Prevalence^2^						
*Polyplastron*						
Before feeding	6.3	28.1	—	0.12	0.57	0.12
6 h after feeding	6.3	3.1				
*Isotricha*						
Before feeding	34.4	43.8	—	0.84	0.84	0.21
6 h after feeding	43.8	31.3				
*Dasytricha*						
Before feeding	21.9	53.1	—	0.88	0.13	0.06
6 h after feeding	34.4	31.3				
*Epidinium*						
Before feeding	6.3	25.0	—	0.77	<0.01	0.42
6 h after feeding	3.1	34.4				

^1^% of total protozoa count.

^2^% of the heifers that had at least one of the following protozoa genus. Data were analyzed as binomial distribution (yes or no) by the GLIMMIX procedure (SAS Inst. Inc.).

### Chewing activity

Total eating, ruminating and chewing time (hours per day) was not affected (*P* ≥ 0.10) by period of measurement (before vs. after rumen transfers; Table 6). However, as a result of higher DMI, heifers spent less time (*P* < 0.01) eating, ruminating and chewing per unit of feed intake (DM or NDF) after rumen transfers.

**Table 6:** Chewing activity of heifers fed a barley straw diet before and after rumen content transfers from the bison.

	Rumen **t**ransfers		SEM	*P*-value
	Before	After		
DMI, kg/d	5.9	7.2	0.13	<0.01
Chewing activity				
Eating activity				
h/d	5.7	5.6	0.11	0.20
Min/kg of DM	58.6	47.6	1.75	<0.01
Min/kg of NDF	88.8	72.1	2.69	<0.01
Ruminating time				
h/d	8.1	8.3	0.18	0.10
Min/kg of DM	82.9	70.2	1.60	<0.01
Min/kg of NDF	125.8	106.4	2.58	<0.01
Total chewing time				
h/d	13.9	13.9	0.18	0.74
Min/kg of DM	141.6	117.8	2.61	<0.01
Min/kg of NDF	214.6	178.5	4.16	<0.01

DM, dry matter; NDF, neutral detergent fiber.

### Alpha-diversity measures

Rumen microbiota became more diverse and exhibited higher evenness as a result of the rumen transfers, as indicated by Chao1, Shannon and Simpson indices (*P* < 0.01; Table 7). Although there was a shift towards pre-transfer richness and evenness levels (Shannon and Simpson indices) on day 27 after the second rumen transfer, complete restoration back to pre-rumen transfer populations was not achieved.

**Table 7:** Alpha-diversity indices of bacterial communities in the ruminal contents of heifers fed a barley straw diet before and after rumen content transfers from the bison.

Alpha diversity index	Bison inoculum	Rumen transfers			SEM	*P*-value^2^
		Before	After (d 1)^1^	After (d 27)^1^		
OTUs	3303.0 ± 220.1	3771.9c	4300.5a	4158.8b	35.78	<0.001
Chao1	8875.0 ± 890.1	11841b	16787a	16785a	371.7	<0.001
Shannon	10.078 ± 0.166	10.179c	10.542a	10.428b	0.0342	<0.001
Simpson	0.9957 ± 0.0006	0.9954b	0.9962a	0.9959ab	0.00018	0.002

^1^Days 1 and 27 after the second rumen transfer.

^2^The *P*-values were adjusted for FDR using Benjamini-Hochberg method^85^. A threshold of *P* < 0.15 was applied to determine the significance. Within a row, means with different superscript are significantly different.

### Bacterial composition of rumen microbiota

Phylogenetic analysis identified 20 phyla within the rumen microbiota, 13 of which had a relative sequence abundance <0.5% of the community, which included Armatimonadetes, Chlamydiae, Chloroflexi, Cyanobacteria, Elusimicrobia, Planctomycetes, SHA-109, Synergistetes, SR1, Tenericutes, TM6, TM7, and Verrucomicrobia. The four most abundant phyla in the rumen were Firmicutes, Fibrobacteres, Bacteroidetes, and Spirochaetae (Table 8). One day after the second rumen transfer, the relative sequence abundances of Fibrobacteres decreased (*P* = 0.14) and Spirochaetae increased (*P* = 0.02), but after 27 days they were not different from pre-rumen transfer levels.

**Table 8:** Phylum-level taxonomic composition of the bacterial communities in the ruminal contents of heifers fed a barley straw diet before and after rumen content transfers from the bison.

Phylum (%)	Bison inoculum	Rumen transfers			SEM	*P*-value^2^
		Before	After (d 1)^1^	After (d 27)^1^		
Firmicutes	44.48 ± 0.45	40.53	41.61	39.05	0.012	0.22
Fibrobacteres	23.09 ± 2.10	25.04a	22.32b	25.34a	0.022	0.14
Bacteroidetes	20.04 ± 1.78	20.29	21.52	20.71	0.004	0.21
Spirochaetae	5.82 ± 0.40	6.13b	7.44a	6.44b	0.006	0.02
Actinobacteria	1.95 ± 0.31	1.78a	1.36b	1.44b	0.001	<0.001
Proteobacteria	1.44 ± 0.16	1.64a	1.43b	1.43b	0.001	0.01
Lentisphaerae	1.29 ± 0.15	1.35	1.39	1.22	0.002	0.44
Others (<0.5%)	1.88 ± 0.08	1.91b	1.79b	2.19a	0.11	<0.001

^1^Days 1 and 27 after the second rumen transfer.

^2^The *P*-values were adjusted for FDR using Benjamini-Hochberg method^85^. A threshold of *P* < 0.15 was applied to determine the significance. Within a row, means with different superscript are significantly different.

Actinobacteria and Proteobacteria decreased (*P* ≤ 0.01) after rumen transfers (day 1) and remained lower after 27 days compared to pre-rumen transfers abundancies. In addition, 27 days after rumen transfers the sum of the phyla with less than 0.5% relative sequence abundances (others) was higher (*P* < 0.001) than pre-rumen transfer levels.

A total of 16 families were identified with relative abundancies >0.5% (Table 9). The three most abundant families were Fibrobacteraceae, Lachnospiraceae, and Ruminococcaceae. The relative abundancies of Christensenellaceae, Prevotellaceae and uncultured Bacteroidales increased (*P* < 0.05) and Lachnospiraceae, Veillonellaceae, BS11 gut group, Coriobacteriaceae and Victivallaceae decreased (*P* < 0.05) 27 days after rumen transfers as compared to pre-rumen transfers levels. Fibrobacteraceae and S24-7 decreased (*P* < 0.10), and Family XIII, Spirochaetaceae and RFP12 gut group increased (*P* < 0.01) one day after the transfers, but these returned to pre-transfers levels after 27 days. Only three families (Ruminococcaceae, Acidaminococcaceae and Rikenellaceae) were not affected (*P* > 0.15) by the rumen transfers.

**Table 9:** Family-level taxonomic composition of the bacterial communities in the ruminal contents of heifers fed a barley straw diet before and after rumen content transfers from the bison.

Phylum	Family (%)	Bison inoculum	Rumen transfers			SEM	*P*-value^2^
			Before	After (d 1)^1^	After (d 27)^1^		
Fibrobacteres	Fibrobacteraceae	23.09 ± 2.10	25.09a	22.47b	25.47a	1.119	0.10
Firmicutes	Lachnospiraceae	15.93 ± 0.52	16.17a	15.61a	14.15b	0.479	0.02
	Ruminococcaceae	16.12 ± 0.80	15.26	16.30	15.46	0.465	0.21
	Christensenellaceae	6.90 ± 0.36	5.22b	5.93a	5.98a	0.272	0.07
	Family XIII	2.48 ± 0.16	1.19b	1.38a	1.12b	0.061	0.006
	Acidaminococcaceae	1.12 ± 0.07	0.93	0.86	0.93	0.039	0.30
	Veillonellaceae	0.61 ± 0.03	0.69a	0.41c	0.56b	0.026	<0.001
Bacteroidetes	Prevotellaceae	7.87 ± 0.99	9.66c	11.32a	10.41b	0.356	0.004
	S24-7	3.82 ± 0.25	4.30a	3.83b	4.41a	0.146	0.008
	Rikenellaceae	2.92 ± 0.23	2.48	2.47	2.42	0.064	0.80
	BS11 gut group	2.85 ± 0.34	1.98a	1.67b	1.51b	0.077	<0.001
	Bacteroidales (uncultured)	0.61 ± 0.14	0.48c	0.59b	0.71a	0.037	<0.001
Spirochaetae	Spirochaetaceae	5.73 ± 0.40	5.79b	7.15a	6.06b	0.006	0.01
Actinobacteria	Coriobacteriaceae	1.74 ± 0.29	1.63a	1.23b	1.28b	0.085	<0.001
Lentisphaerae	Victivallaceae	0.40 ± 0.06	0.57a	0.57a	0.39b	0.048	0.01
	RFP12 gut group (uncultured)	0.65 ± 0.08	0.46b	0.60a	0.44b	0.032	0.001
Others (<0.5%)		7.18 ± 0.36	6.90a	6.49b	6.70ab	0.147	0.04

^1^Days 1 and 27 after the second rumen transfer.

^2^The *P*-values were adjusted for FDR using Benjamini-Hochberg method^85^. A threshold of *P* < 0.15 was applied to determine the significance. Within a row, means with different superscript are significantly different.

**Figure 2:**
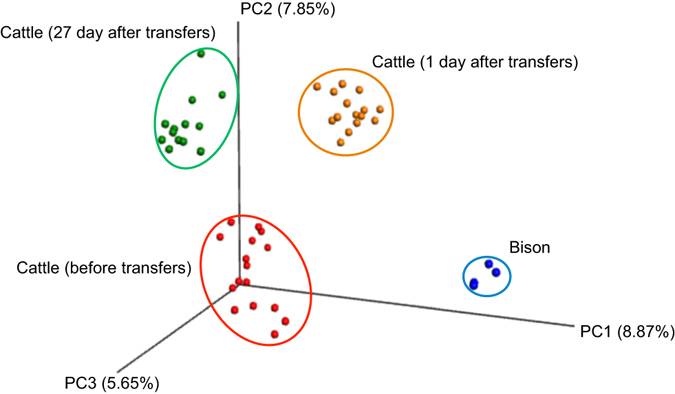
Principal coordinate analysis (PCoA) of ruminal bacterial OTUs from cattle before rumen content transfers and after transfers (days 1 and 27), and from the second bison inoculum used in the transfers. Proportion of variance explained by each principal coordinate axis is denoted in the corresponding axis label.

Principle coordinate analysis of the Bray-Curtis similarity metric clearly showed that the microbial community in the cattle prior to transfer was distinct from the community after transfer (Fig. 2). The rumen microbial community of the bison was distinct from the cattle both before and after transfers. There was a trend for the cattle rumen community to shift back towards the pre-transfer structure, but it did not return to pre-transfer levels within 27 days.

### Correlation between digestibility variables and rumen microbiota

A Pearson correlation matrix was created to evaluate the relative impact of numbers and types of rumen protozoa and sequence abundance of the bacterial families with diet digestibility parameters (Table 10). Diet DM digestibility was positively correlated with *Eudiplodinium* (Pearson correlation coefficient (r) = 0.34, *P* < 0.05) and *Metadinium* (r = 0.44, *P* < 0.01). *Metadinium* was also strongly positively correlated with diet NDF (r = 0.54, *P* < 0.01) and ADF (r = 0.50, *P* < 0.01) digestibility. Apparent N digestibility was positively correlated with total rumen protozoa (r = 0.42, *P* < 0.05) and the relative sequence abundance of Christensenellaceae (r = 0.37, *P* < 0.05). Apparent N digestibility was negatively correlated with the relative sequence abundance of the BS11 gut group (r = −0.46, *P* < 0.01). The abundance of Ruminococcaceae was negatively correlated with Fibrobacteraceae (r = −0.63, *P* < 0.001) and positively correlated with Prevotellaceae (r = 0.47, *P* < 0.01).

**Table 10:** Pearson correlation coefficients between diet digestibility variables, total protozoa, and relative taxa abundances of ruminal protozoa and bacteria.

	DM digestibility	NDF digestibility	ADF digestibility	N digestibility	Ruminococcaceae
Total protozoa				0.42*	
*Eudiplodinium*	0.34*				
*Metadinium*	0.44**	0.54**	0.50**		
Christensenellaceae				0.37*	
BS11 gut group				−0.46**	
Fibrobacteraceae					−0.63***
Prevotellaceae					0.47**

^*^ *P* < 0.05; **P < 0.01; ***P < 0.001.

## Discussion

To investigate if members of the bison rumen microbiome could occupy favourable niches and potentially complement those members of the community in cattle responsible for fiber degradation, we transferred bison rumen contents into the rumen of heifers fully adapted to a barley straw diet. A previous study that exchanged rumen contents between cows maintained on the same diet demonstrated that the host animal quickly re-establish its rumen microbiome after transfer^19^. Consequently, we decided to conduct the bison rumen content transfer twice to increase the likelihood that microbes associated with the degradation of the most recalcitrant fibre had the opportunity to establish in the rumen of heifers. As our intent was not just to modify the rumen microbes, but to try to make the population even more efficient than what it was before transfer, 30% of the heifers’ ruminal content wet weight was returned to the rumen of the original host.

Rumen content transfer across ruminant species may be a means of overcoming the problem of reduced environmental fitness promoted by laboratory culture methods. Transplantation of rumen contents has been successfully used to clinically treat indigestion and return the rumen to its normal function, and to convert toxic compounds found in some plants to harmless or even beneficial compounds^20^. The exchange of ruminal contents from non-fasted to fasted lambs has also been suggested to accelerate repletion of the protozoal population after re-alimentation^21^.

Studies on the transfer of rumen contents have also proven this technique to be an effective way to colonise protozoa-free rumens with specific protozoa species^16, 22, 23^. Limited information exists on the role of rumen protozoa in fiber degradation^24, 25^, but functional protozoal glycosyl hydrolases have been identified, successfully expressed and biochemically characterized^26^. Rumen fiber degradation depends on complex interactions between bacteria, protozoa and fungi and the plant cell wall. The specific contribution of rumen protozoa to fiber degradation in the rumen of cattle fed high forage diets at low intakes has been modeled to be between 17 and 21%^27^. Recent studies have shown that fiber digestibility in sheep fed forage diets declined anywhere from 14 to 17% in protozoa-free as compared to faunated sheep^17, 18^. Protozoa may also stimulate fiber degradation in the rumen through indirect mechanisms. Ruminal ciliates are able to utilize low concentrations of O_2_, stabilizing ruminal metabolism^28, 29^. They also host epi- and endo-symbiotic methanogens that are protected from O_2_ toxicity^30^. Methanogens are strict anaerobes that through interspecies H_2_ transfer, utilize the H_2_ produced by fibrolytic microorganisms to reduce CO_2_ to CH_4_^31^. Rumen ciliates are also involved in the initial stages of colonization and digestion of fiber^13, 32^. A recent meta-analysis showed that defaunated ruminants possessed a lower concentration of fibrolytic microbes including anaerobic fungi (−92%), *R*. *albus* (−34%) and *R*. *flavefaciens* (−22%). This was linked to lower fiber digestibility (−11%) and methane production (−11%)^32^, demonstrating the important contribution that protozoa make to ruminal fiber degradation.

The high pH (>7.0) of the bison rumen contents used for the rumen transfers is most likely a reflection of the high forage diet (75:25 barley silage:oats diet, DM basis) along with fasting for ~12 h prior to slaughter. In the present study, the DMI (kg/d) increased over the duration of the experiment. However, in contradiction to our hypothesis, total diet and fiber digestibility were not improved by rumen transfers. If intake increased, with no change in the fractional passage or digestibility of digesta as a result of rumen transfers, the mass of rumen contents must have also increased. This is supported by the increase in total DM in the gut (% of BW) calculated after rumen transfers. The reticuloruminal capacity seems to have increased at a rate faster than that of BW because intake as % of BW and as g per kg of BW^0.75^ was also higher after transfer of rumen contents. Increase in the reticulorumen as a proportion of empty BW and in reticulorumen contents in ruminants fed low-quality forage compared with high-quality forage have been previously observed^33–36^. In the present study, heifers fed a barley straw diet most likely increased the mass of the reticulorumen as a proportion of empty BW as a way to increase total digestible energy intake in an effort to meet energy demand. As we did not have a control group that was not subjected to rumen content transfer, it is not possible to say if this increase in intake was due to the rumen content transfers, or the advancing age of the heifers and physiological adaptation to the diet.

Previous studies with faunated and protozoa-free ruminants have documented that the role of protozoa in feed protein degradation becomes more important as diet protein solubility decreases^37, 38^. The increase in diet N digestibility as a result of the bison rumen content transfer is consistent with the increase in total protozoa counts. The present study is in agreement with these previous observations and shows the importance of protozoa in feed protein degradation when heifers were fed a diet with low protein solubility. In addition to protozoa, the Pearson correlation analysis indicated that the increase in N digestibility was also associated with an increase in the relative sequence abundance of Christensenellaceae and a decrease in the uncultured BS11 group. Members of the Christensenellaceae are anaerobic, gram-negative rod shaped cells with this family only being recently described^39^. One of its members, *Christensenella minuta*, has been shown to produce acetic and butyric acids as end products from the fermentation of glucose^39^, but little is known about its function within the gut microbiome. An interesting study by Goodrich *et al*.^40^, reported that Christensenellaceae were enriched in the gut of humans with low body mass index, and inoculation of germfree mice with *C*. *minuta*, reduced weight gain and caused shifts in the murine gut microbiome. These results confirm that Christensenellaceae abundance has an important role in regulating the gut microbiome. Recent metagenomic analysis of the uncultured BS11 gut group revealed their role in hemicellulose degradation, producing butyrate and acetate as end products of fermentation in the rumen^41^. The BS11 group was shown to be enriched in the rumen of Alaskan moose fed winter diets which are lower in protein and higher in hemicellulose and lignin as compared to spring and summer diets^41^. In the present study, the negative relationship between N digestibility and the relative sequence abundances of uncultured BS11 may suggest that this group is adapted to conditions of lower N availability in the rumen.

Although N intake and digestibility were increased, the efficiency of microbial N production (g/kg of digested OM) was not affected, and retained N as percentage of N intake or N digested only showed a tendency to increase. The low digestible energy content of the diet may have limited the availability of carbon skeletons for microbial protein synthesis and also stimulated the hepatic catabolism of amino acids as an energy source, leading to an increase in urinary N excretion.

Transfer of rumen contents from bison to cattle was an effective way of increasing the total number and diversity of the protozoa population. The increase in total protozoa numbers was mainly attributable to *Ostracodinium*. *Ostracodinium* grows on ground straw and possesses one of the highest concentrations of cellulases and β-glucosidases of the rumen ciliates^13^. The disturbance of the rumen environment caused by the rumen transfers associated with the high fibrous barley straw diet fed to the heifers may have created favorable conditions for the establishment and growth of *Ostracodinium*, compared to other rumen ciliates.

The small reduction in ruminal pH and the increase in NH_3_-N and total VFA after rumen transfers are consistent with an increase in DMI and N digestibility. Increase in ruminal NH_3_-N concentration seems also to be associated with higher numbers of protozoa in rumen contents, as a result of the proteolytic activity of these eukaryotes against bacteria^17, 42^ and their role in the degradation of feed protein^43^. Ruminal oxidative-deamination and decarboxylation of valine, leucine, and isoleucine are the primary source of branched-chain VFA in the rumen^44^. Hence, the higher molar proportions of the branched-chain VFA (valerate, isovalerate and isobutyrate) are most likely a reflection of the higher feed protein degradation and turnover of bacterial protein in the rumen as a result of increased protozoa numbers after rumen transfers. The major fiber degrading bacteria (i.e. *R*. *albus*, *R*. *flavefaciens*, *F*. *succinogenes*, and *Butyrivibrio fibrisolvens*) require branched-chain VFA for growth^45–47^. In addition, some studies also observed that ruminal supplementation with branched-chain VFA can improve ruminal fiber degradation *in vitro* and *in vivo* ^46, 48–50^. Although branched-chain VFA increased after rumen transfers, fiber digestibility was not improved, suggesting that branched-chain VFA concentrations were not limiting fiber degradation. The increased proportion of butyrate seems also to be related to the increase in the numbers of protozoa because butyrate and acetate are the main VFA produced as the result of fermentation of starch and cellulose by protozoa^13, 51, 52^. Increased butyrate formation may also be a result of an increase in acetate to butyrate conversion in the rumen via butyryl CoA:acetate CoA transferase^53–55^. Sutton *et al*.^55^ labeled VFA with ^14^C and found that up to 64% of the carbon in butyrate originated from acetate. The small reduction in the proportion of acetate, and consequently in the C2:C3, is likely a result of the increased conversion of acetate to butyrate as well as rumen microorganisms using acetate for amino acid biosynthesis.

Total daily eating, ruminating and chewing (eating + ruminating) activity (h/d) were not affected by rumen transfer. However, as DMI increased, the eating, ruminating and chewing time required per unit of DM or NDF (min/kg of DM or NDF) decreased. Eating, ruminating and total chewing time per kg of cell wall constituents have been shown to be negatively correlated to age and BW of heifers^56–58^. This is consistent with the present study because heifers were growing and were heavier in the period after rumen content transfers. Heifers appeared to have reached their maximum daily ruminating time (~8 h) as previously documented by Welch^56^, where the maximum time spent ruminating was 8 to 9 h/d for most ruminants. Interestingly, although chewing time per kg of DM or NDF were reduced after rumen transfer, diet digestibility was not negatively affected. This seems to be a result of more efficient bites in older heifers promoting more effective comminution of the ingested feed^59^. More efficient mastication as heifers age is consistent with the developmental stage of the teeth^58, 60^.

## Conclusion

Repeated inoculation of the rumen of cattle with bison rumen contents successfully altered the rumen microbiome in cattle. The relative numbers of protozoa and sequence abundance of several families of bacteria were altered as a result of rumen content exchange and these changes were maintained over time. Furthermore, transfer of rumen contents increased protein digestibility and nitrogen retention and altered the proportion of VFAs. However, in contradiction to our hypothesis DM and NDF digestibility in cattle was not improved by the inoculation of the rumen with bison rumen contents. Future studies should explore the potential for direct transfer of rumen contents with enhanced anaerobic fungal populations to improve the digestion of recalcitrant forages in cattle.

## Materials and Methods

### Ethics Statement

All procedures and protocols used in this study were reviewed and approved by the Animal Care Committee at the Lethbridge Research and Development Centre of Agriculture and Agri-Food Canada. Care and management of heifers followed the guidelines of the Canadian Council on Animal Care^61^.

### Experimental design, animals, housing, diet and measurement procedures

The experiment was a repeated measure design with one treatment (rumen transfers) that had two levels (before and after rumen transfers), with 16 heifers as experimental units. The study was conducted using rumen fistulated Angus × Hereford cross beef heifers [461 ± 21 kg body weight (BW); age = 14 ± 2 months]. The heifers were housed in tie stalls on rubber mats bedded with wood shavings for the 18 week study and were exercised for 2 h daily in an open drylot. Before the start of the study, heifers were treated with 1% w/v ivermectin (Ivomec®, Merial Canada Inc., Baie D’Urfé, Quebec, Canada).

**Figure 3:**
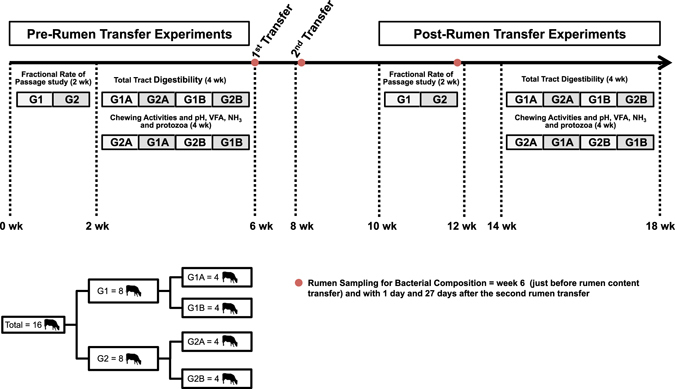
Experimental layout. The 16 heifer were divided in 2 groups of 8 animals of similar average body weight (G1 and G2) for the fractional rate of passage study. These 2 groups were further sub-dived in 4 groups of 4 heifers each with similar average body weight (G1A, G1B, G2A and G2B) for the other measurements. Rumen sampling for Bacterial composition were conducted in all the heifers in the same days.

Heifers were fed a barley straw diet consisting of 70:30 forage-to-concentrate [dry matter (DM) basis; Table 11] formulated to meet or exceed protein, mineral and vitamin requirements of beef heifers weighing 450 kg and gaining 1.0 kg/d^62^. The amount of concentrate (protein supplement) fed to each heifer was calculated as 30% of total DM intake of the previous week (7 days). Barley straw was chopped to 6 to 10 cm and the concentrate was fed at 0930 h. Heifers were allowed 30 min to consume concentrate before being offered barley straw. Barley straw was provided *ad libitum* so as to ensure 10 to 20% orts. On the beginning of each week, total DM intake from the previous week was calculated for each heifer and the amount of concentrate provided to each heifer was adjusted as described above. Heifers were adapted to the barley straw diet for 28 days prior to the start of the 126 day experiment (18 weeks). Intake, chewing activity, ruminal fractional rate of passage of fluids and solids, total tract digestibility, rumen fermentation, and the bacterial and protozoal communities were examined at defined periods as outlined in Fig. 3. Forty-six days after the start of the experiment (on week 6), ~70% of rumen contents were removed from each heifer and replaced with mixed rumen contents collected immediately after the slaughter of 32 bison. This entire procedure was repeated a second time 14 d later. Bison were maintained on a mixed grass pasture prior to being finished for 90 days using a 75:25 barley silage:oats diet (DM basis). Bison were approximately 24 months of age at slaughter.

**Table 11:** Chemical composition of the barley straw and concentrate fed to heifers and diets consumed during each period.

Chemical composition	Barley straw^1^	Concentrate^2^	Diet consumed before transfers^3^	Diet consumed after transfers^3^
DM, %	92.0 ± 0.89	92.4 ± 0.41	92.5 ± 0.01	92.5 ± 0.01
OM, % of DM	92.8 ± 1.59	90.1 ± 0.36	92.0 ± 0.07	92.0 ± 0.03
CP, % of DM	6.3 ± 1.17	38.3 ± 0.37	15.2 ± 0.82	15.8 ± 0.33
NDF, % of DM	78.0 ± 3.89	37.6 ± 1.41	66.7 ± 1.04	66.0 ± 0.41
ADF, % of DM	46.6 ± 3.46	16.0 ± 0.65	38.1 ± 0.79	37.6 ± 0.31

DM, dry matter; OM, organic matter; CP, crude protein; NDF, neutral detergent fiber; ADF, acid detergent fiber.

^1^Fed to achieve 70% of diet DM.

^2^Fed to achieve 30% of diet DM. Composition (DM basis): 66.7% of corn dried distillers grains with solubles, 26.6% of canola meal, 4.2% calcium carbonate, 1% of urea, 0.8% dicalcium phosphate, 0.5% salt, 0.17% feedlot premix, and 0.01% vitamin E. Feedlot premix supplied per kg of diet DM: 65 mg of Zn, 28 mg of Mn, 15 mg of Cu, 0.7 mg of I, 0.2 mg of Co, 0.3 mg of Se, 6000 IU of vitamin A, 600 IU of vitamin D, and 47 IU of vitamin E.

^3^Diet consumed during the 5 days total collection period.

To measure the ruminal fractional rate of passage, heifers were blocked by weight and divided into 2 groups of 8, and measurements were obtained from each group in consecutive weeks (one group per week; Fig. 3). For other measurements, the two groups of heifers were further divided into sub-groups of 4 heifers and measurements were obtained as outlined in Fig. 3, both before the first rumen transfer and 14 d after the second rumen transfer. Full body weight was recorded just before feeding every 4 weeks and on 2 consecutive days at the beginning and the end of the study to determine gain or loss of BW.

### Rumen transfers

The purpose of two rumen transfers was to increase the likelihood that microbes associated with the degradation of the most recalcitrant fibre (bison inoculum) had the opportunity to establish in the rumen of the heifers. Whole bison rumens (n = 32) were collected at a commercial abattoir immediately after slaughter with the end of the esophagus and the junction between the pylorus and the small intestine sealed using plastic zip ties. Rumens along with the omasum and abomasum were placed in large plastic bags inside an insulated container and immediately transported to the Lethbridge Research and Development Centre (LRDC) in a heated truck. At the metabolism barn, bison rumens were opened and the contents were poured into a holding tank, mixed and maintained at 39 °C and continuously flushed with O_2_-free CO_2_. The rumen contents from each heifer were completely evacuated just before feeding and placed inside a sealed insulated container and weighed. Contents were mixed and 30% of the wet weight was returned to the rumen of the individual from which it was collected to ensure that the ruminal microbes that were adapted to the degradation of barley straw were retained. As a result, pooled bison rumen contents replaced 70% of contents within the rumen of cattle. Immediately before the rumen transfer, samples of rumen contents from individual heifers and the pooled bison rumen contents were collected for subsequent analysis of chemical composition, pH, VFA concentration, NH_3_-N concentration, and protozoa. The entire rumen transfer procedure was repeated a second time 14 days later, using another 32 bison rumens that were from the same source as previously described. Samples of rumen contents from individual heifers and the pooled bison rumen contents were also collected immediately before the second rumen transfer for analysis as described above. All rumen transfers were completed within 4 h from the time of initial collection of bison rumen contents at the slaughter plant.

### Feed intake

Individual intakes were recorded daily throughout the study by weighing the amount of feed offered and amount of feed refused. Samples from the feed and orts were collected weekly. During the period to estimate total tract digestibility, daily samples of feed offered and orts were collected and pooled by animal and by period. Samples from the feed and orts were dried in an oven at 55 °C for 72 h and used to determine DM, organic matter (OM), nitrogen (N), neutral detergent fiber (NDF), and acid detergent fiber (ADF) intake.

### Chewing activities

Chewing behavior was monitored using 4 video cameras (WV-CP474; Panasonic, Mississauga, ON, Canada) with vari-focal lens (2.8–12 mm; Tamron Co. Ltd., Saitama City, Japan). Each camera was positioned in front of the heifer tie stalls (1.73 m off the floor and 2.44 m from the tie rail). Video was recorded for 5 days (24 h/d) during each period onto 1 of 4 time-lapse video cassette recorders (AG-RT650 Panasonic) connected to the cameras. The videotapes from the 5 days of each period were viewed and summarized by one trained observer. The chewing activity (eating and ruminating) of each heifer was recorded for each minute of the day. Chewing activities were expressed as total hours for the 24-h period and on the basis of DM and NDF intake by dividing minutes of eating or ruminating by intake^63^.

### Total tract digestibility

Apparent total tract digestibility of nutrients was estimated by total collection of urine and feces for 5 days. Heifers were fitted with urinary catheters (Bardex Lubricath Foley catheter, 75 c.c. and 26 Fr.; Bard Canada Inc., Oakville, ON, Canada) to ensure separate collection of urine and feces. Loss of NH_3_ from urine was prevented by acidifying urine with 4 *N* H_2_SO_4_ so that urine pH remained <2 during collection. Total output of urine and feces were measured every 24 h and samples were thoroughly mixed and subsampled. An aliquot of the daily urine was diluted with distilled water at a ratio of 1:5 to prevent precipitation of uric acid^64^ and stored at −20 °C until analyzed for total N and purine derivatives. A sample (≈500 g) of the feces pooled over 5 days was dried for 72 h at 55 °C in an oven to a constant weight. The fecal samples were analyzed for DM, ash, N, NDF, and ADF. Urine samples were also dried at 55 °C before N analysis. Apparent digestibility of nutrients was calculated by the difference between intake and fecal output of the nutrient. Retention of N was calculated by the difference between digested N and urinary N output. Ruminal microbial protein synthesis was estimated by measuring allantoin and uric acid in urine as described by Chen and Gomes^64^. Total microbial purine absorption (mmol/day) was calculated as:

$${\rm{Purine}}\,{\rm{absorption}}=({\rm{total}}\,{\rm{PD}}\,{\rm{excretion}}-0.385\times {{\rm{BW}}}^{75})/0.85$$

where total PD excretion = total purine derivatives excretion in mmol/day; and 0.85 is the efficiency of absorption of purines^64^.

Ruminal microbial protein synthesis (g/day) was calculated as:

$${\rm{microbial}}\,{\rm{N}}\,=({\rm{purine}}\,{\rm{absorption}}\times 70)/(0.116\times 0.83\times 1000)$$

where purine absorption is in mmol/day; 70 is the N content of purines (mg N/mmol); 0.116 is the ratio of purine-N:total-N for mixed rumen microbes; and 0.83 is the digestibility of microbial purines^64^.

### Digesta kinetics and gut fill

Ytterbium-labeled NDF (Yb-NDF) was used as a marker for fractional solid passage rates. Barley straw was cut into 6-cm lengths, boiled (80 °C for 1 h) using a commercial detergent without EDTA to remove cell solubles, and labelled with Yb as described by Mambrini and Peyrand^65^. The fractional passage rate of fluids was measured using LiCo-EDTA as a marker as described by Uden *et al*.^66^. Heifers received a single dose of 146 g of Yb-NDF and 15 g of LiCo-EDTA via the cannula at the time of the morning feeding. The Yb-NDF was placed in the rumen and LiCo-EDTA was administered using a plastic funnel connected to a flexible tube directly into the ventral sac of the rumen. After dosing the markers no attempts were made to manually mixture the markers with the rumen contents as described by Zebeli *et al*.^67^. Fecal grab samples were taken at 0 (before dosing and immediately before feeding), 4, 8, 12, 16, 20, 24, 32, 40, 48, 60, 72, 84, 96, 120, and 144 h after dosing to determine the fractional passage rates of digesta. Samples were dry-ashed and fecal marker concentrations of Yb and Co were determined using Inductively Coupled Plasma Emission Spectroscopy according to the AOAC^68^, but without CaCl_2_ during sample digestion.

Fecal Yb and Co excretion curves were fitted to a 2-compartment model, both as age-independent and age-dependent models, as described by Moore *et al*.^69^. These models estimate fractional passage rates from 2 compartments [slow (k_S_, passage out of the rumen) and fast (k_F_, lower digestive tract)] and include a time delay. Total mean retention time in the digestive tract (TMRT) was calculated as time delay plus the sum of the mean retention time in the rumen (RMRT) and in the lower digestive tract (FMRT). Data were analyzed by the NLIN procedure (iterative Marquardt method) of SAS (SAS Institute Inc., Cary, NC, USA). The criteria of Moore *et al*.^69^ were used to identify the best-fit model and the nonlinear model with gamma-4 age dependency in the fast compartment (G4G1) was selected.

The total DM present in gut was calculated using the following equation which assumes a linear absorption of nutrients during TMRT of solids^70^:

$${\rm{Total}}\,\mathrm{DM}\,\mathrm{in}\,\mathrm{gut}={\rm{DMI}}\times [1-({\rm{DMD}}/2)]\times {\rm{TMRTs}}$$

where DMI = dry matter intake (kg/h); DMD = apparent total tract DM digestibility; and TMRTs = total mean retention time in the digestive tract of the solids (h).

### Ruminal sampling for VFA, NH_3_-N, protozoa and pH measurements

Ruminal contents were collected from the reticulum, ventral, caudal and dorsal-ventral sac of the reticulo-rumen of each heifer prior to the morning feeding and at 6 h after feeding. Prior to the morning feeding and just after sampling of ruminal contents, an indwelling LRC pH meter (Dascor, Inc., Escondido, CA) was inserted into the rumen and ruminal pH was recorded every minute for 5 days. The pH data logger was retained in the ventral sac by a 0.5 kg sealed stainless steel weight and anchored by 60 cm of cable connected to the ruminal cannula plug. Prior to insertion, electrodes were calibrated using pH 4 and 7 buffers. Ruminal pH data were summarized for daily average, minimum, maximum and standard deviation (SD) of mean pH. Ruminal contents were sampled again 5 days later prior to the morning feeding and at 6 h after feeding upon removal of the pH meters. This same sampling procedure described above was repeated after the second rumen transfer.

Rumen contents from each heifer were combined, thoroughly mixed and strained through two layers of PECAP nylon (pore size 355 μm; Sefar Canada Inc., Ville St. Laurent, Canada). After pH was measured, samples of the collected fluid (5 mL) were mixed with 1 mL of 25% (wt/vol) metaphosphoric acid for VFA analysis, with 1 mL of 1% H_2_SO_4_ for NH_3_-N determination. An additional 5 mL of rumen fluid was mixed with 5 mL methyl green formalin-saline solution for later enumeration and identification of protozoa. Samples were stored at −20 °C until analyzed for VFA and NH_3_-N. Protozoa samples were stored in the dark at room temperature until examined. Protozoa were enumerated and genera identified by light microscopy using a Levy-Hausser counting chamber (Hausser Scientific, Horsham, PA, USA) as described by Dehority^71^. Each sample was enumerated in duplicate and the average value was used for data analysis. If the average of the duplicates differed by more than 10%, counts were repeated.

### Chemical analysis

Feed, orts and fecal samples were ground to pass a 1-mm screen (standard model 4 Wiley mill, Arthur H. Thomas, Philadelphia, PA, USA) for chemical analysis. Subsamples (5 g) were further ground using a ball grinder (Retsch MM 400, Newtown, PA, USA) and analyzed for N by flash combustion (Carlo Erba Instruments, Milan, Italy). Crude protein was calculated as N × 6.25. Urine samples were also analyzed for N as described above. Both NDF and ADF were determined by the sequential method with the F57 ANKOM filter bag (pore size 25 μm) and ANKOM200 Fiber Analyzer (ANKOM Technology, Macedon, NY, USA) using reagents as described by Van Soest *et al*.^72^, with the addition heat-stable α-amylase and sodium sulfite in the NDF procedure and expressed inclusive of residual ash. Ash content was determined by combustion of samples in a muffle furnace at 550 °C for 5 h.

Ruminal VFA concentrations were quantified by gas chromatography (model 5890A Series Plus II, Hewlett Packard Co., Palo Alto, CA, USA). The chromatograph was equipped with a 30-m Zebron free fatty acid phase fused silica capillary, 0.32-mm i.d. and 1.0-μm film thickness column (Phenomenex, Torrance, CA, USA). For VFA 0.1 M crotonic acid was used as an internal standard, as described by Bevans *et al*.^73^. The NH_3_-N concentration in rumen fluid was analyzed by the phenol-hypochlorite method as described by Broderick and Kang^74^.

Uric acid in urine samples was determined by a colorimetric procedure using a commercial kit (MAK077, Sigma-Aldrich Co., St. Louis, MO, USA) and allantoin in urine samples was determined by a colorimetric method as described by Young and Conway^75^.

### Rumen content sampling for RNA extraction and bacterial composition

Rumen content samples were obtained in the morning, immediately before feeding, before heifers received the first rumen inoculum transfer, and after 1 and 27 days of the second rumen inoculum transfer. Rumen contents from bison used as inoculum in the second rumen transfer was also sampled for subsequent analysis. The samples were transferred to 250 ml beakers and the solid and liquid phases were separated using a Bodum coffee filter plunger (Bodum Inc., Triengen, Switzerland). Subsamples of solid digesta (~5.0 g) were immediately flash-frozen in liquid N and stored at −80 °C until further processing.

Total RNA was isolated from rumen solids according to Wang *et al*.^76^. Total RNA quality was verified by running the samples on RNA 6000 nano chip (Agilent Technologies, Mississauga, Ontario, Canada) on an Agilent 2100 BioAnalyzer (Agilent Technologies).

### PCR amplification and 16S rRNA aomplicon sequencing

Bacterial 16S rRNA genes were amplified using the primers Bact_341F (5′-TATGGTAATTGTACTCCTACGGGNGGCWGCAG-3′) and Bact_806R (5′-AGTCAGTCAGCCGGACTACHVGGGTATCTAAT-3′)^77, 78^. The dual barcode assay adapted for the Illumina MiSeq sequencer (Illumina Inc., San Diego, USA) was used. Each primer contained the Illumina adapter sequence, unique barcode, spacer and forward or reverse primer. For each cDNA sample, 20 μL of reaction mix was prepared containing 1 μL cDNA, 1 μL of each barcoded primer (1 μM), 7 μL of molecular biology grade H_2_O, and 10 μL of KAPA2G Robust Hotstart ReadyMix (Kapa Biosystems, Wilmington, USA). The PCR reaction conditions were as follows: initial denaturation at 95 °C for 5 min; 20 cycles of denaturation (95 °C, 20 s), annealing (55 °C, 15 s) and elongation (72 °C, 5 min); and a final 10-min extension at 72 °C. Each cDNA sample was amplified in duplicate, and 3 wells per run served as a negative control for the master mix. After amplification, duplicate PCR products were pooled, and the correct sizes of PCR products and the absence of signal from negative controls were further verified through agarose gel electrophoresis. Quantitation of amplicons was performed in a Synergy HTX Multi-Mode Microplate Reader (model SIAFRM, Bio-Tek Instruments Inc., Winooski, USA) using a Quant-iT dsDNA Assay Kit (Thermo Fisher Scientific, Waltham, USA). The amplicons were pooled in equimolar concentrations and purified using Agencourt AMPure XP beads (Beckman Coulter Inc., Brea, USA) and then further quantified as described above. The amplicon library was combined with 10% PhiX control library and sequenced in the Illumina MiSeq (Illumina Inc., San Diego, USA).

### Bacterial diversity analysis

The quality of reads in the raw fastq files from the MiSeq were checked using the program FastQC^79^. Raw reads with an average quality score <20 over a 4 bp sliding window and reads with lengths shorter than 36 bp were removed using Timmomatic v0.33^80^. Paired end reads were merged with PEAR v0.9.8 using default parameters^81^ and unassembled reads were discarded. The remaining merged, high quality reads were used for sequence analysis, OTU detection, taxonomic assignment and phylogenetic analysis with QIIME 1.9^82^. The sequences were clustered into Operational Taxonomic Units (OTUs) using the *de novo* OTU picking workflow within QIIME using a 97% similarity threshold.

Alpha and Beta diversity metrics were calculated using QIIME. Alpha-diversity of samples was assessed by comparing Chao1, Shannon, and Simpson metrics, the number of observed OTUs, and the taxonomic abundance. Sequences were subsampled to the lowest number of sequences found in all samples (9,888 reads) to ensure alpha- and beta-diversity analysis used the same number of sequences per sample. The beta diversity of the samples was compared using the Bray-Curtis dissimilarity index^83^. Principal coordinate analysis of Bray-Curtis dissimilarity indices was carried out and the differences in community structure were visualized with a three dimensional principal coordinate analysis (PCoA) plot.

### Statistical Analysis

Data were analyzed using the MIXED procedure (SAS Inst. Inc., Cary, NC) with period (before the rumen transfers and after the second rumen transfer) included as fixed effect and heifers as random effect. Period was treated as repeated measures to account for possible correlations between the two measurements collected on each animal. Total protozoa and the *Entodinium*, *Ostracodinium, Eudiplodinium, Metadinium* and *Diplodinium* populations were also analyzed as a repeated measures design, but with transfer, sampling time (before feeding and 6 h after feeding) and the transfer × time interaction included as fixed effects in the model. The GLIMMIX procedure (SAS Inst. Inc.) was used to analyze the prevalence (% of the heifers that had at least one of the specific protozoa genus) of the *Polyplastron, Isotricha, Dasytricha, Epidinium* populations. Differences were declared significant at *P* < 0.05.

To compare the changes in bacterial abundance as a result of rumen transfer, the relative sequence abundance of bacterial phyla was arcsine-square-root transformed^84^, and then analyzed as repeated measures with a model similar to the described above to compare the differences among sampling days [before transfers (day 0) and after the second transfers (days 1 and 27)]. Comparison of bacterial sequence abundance at the family level was subjected to a similar repeated measures analysis, but because it was normally distributed, transformation was unnecessary. False discovery rate (FDR) corrected *P*-values were calculated using the Benjamini-Hochberg method^85^, and differences in bacterial taxa abundance were declared significant at FDR-corrected *P*-value < 0.15^86^.

Pearson correlation coefficients between diet digestibility variables, total protozoa, and relative taxa abundances of ruminal protozoa and bacteria were analyzed using the PROC CORR procedure of SAS.
